# On the Causes of Idiocy

**Published:** 1858-07-01

**Authors:** S. G. Howe

**Affiliations:** Of Massachusetts, U.S.A.


THE JOURNAL

OF

PSYCHOLOGICAL MEDICINE

AND

MENTAL PATHOLOGY.

JULY 1, 1858.

### ART. I.—ON THE CAUSES OF IDIOCY.

BY S. G. HOWE, M.D.,

*Of Massachusetts, U. S. A.*

From a Report of the Commissioners appointed by the Legislature of Massachusetts, in 1846, to inquire into the Condition of the Idiots of the Commonwealth.

ALL those who have a living and abiding faith and trust in the goodness and wisdom of the Creator, will readily believe that the terrible evils which now infest society are not necessarily perpetual; that they are not inherent in the very constitution of man, but are the chastisements sent by a loving Father to bring back his children to obedience to his beneficent laws. These laws have been as much shrouded in darkness, in times past, as the hieroglyphics of Egypt; and though they were written upon every man's body, no Champollion was found to decipher them. But a better day has dawned, and men are beginning to read the handwriting upon the world, which tells them that every sin against a natural law must be atoned for by suffering *here* as well as hereafter.

It is beginning to be seen, also, that man has a double nature and double interests; that he is a social being, as well as an individual; and that he cannot sin with impunity against the one nature any more than he can against the other. God has joined men together, and they cannot put themselves asunder. The ignorance, the depravity, the sufferings of one man, or of one class of men, must affect other men, and other classes of men, in spite of all the barriers of pride and selfishness which they may erect around themselves. The doctrine of impenetrability does not obtain in morals, however it may do in physics; but, on the contrary, as gases afford mutually a vacuum to each other into which they rush, so the nature of every individual is a vacuum to the nature of society, and its influences, be they for good or be they for evil, interpenetrate him in spite of himself. It is clear, therefore, that in this, as in everything else, the interest and the duty of society are common and inseparable.

Idiocy is a fact in our history of momentous import. It is one of the many proofs of the immense space through which society has yet to advance before it even approaches to the perfection of civilization which is attainable. Idiots form one rank of that fearful host which is ever pressing upon society with its suffering, its miseries, and its crimes, and which society is ever trying to hold off at arm's length—to keep in quarantine, to shut up in gaols and almshouses, or, at least, to treat as a parish caste; but all in vain.

There are the paupers—a host in themselves; the drunkards, the vagabonds, the criminals, the insane, the blind, the deaf—all these together form a number, the proportion of which to the whole population is fearfully great, and the existence of which is a reproach to our civilization, for that existence implies gross ignorance and open violation of the laws of nature.

The moral to be drawn from the existence of the individual idiot is this:—he, or his parents, have so far violated the natural laws, so far marred the beautiful organism of the body, that it is an unfit instrument for the manifestation of the powers of the soul. The moral to be drawn from the prevalent existence of diocy in society is, that a very large class of persons ignore the conditions upon which alone health and reason are given to men, and consequently they sin in various ways; they disregard the conditions which should be observed in intermarriage; they overlook the hereditary transmission of certain morbid tendencies, or they pervert the natural appetites of the body into lusts of divers kinds—the natural emotions of the mind into fearful passions—and thus bring down the awful consequences of their own ignorance and sin upon the heads of their unoffending children.

Idiocy is found in all civilized countries, but it is not an evil necessarily inherent in society; it is not an accident; and much less is it a special dispensation of Providence; to suppose it can be so, is an insult to the Majesty of Heaven. No! It is merely the result of a violation of natural laws, which are simple, clear, and beautiful; which require only to be seen and known, in order to be loved; and which, if strictly observed for two or three generations, would totally remove from any family, however strongly predisposed to insanity or idiocy, all possibility of its recurrence.

No scientific exposition of these laws will be attempted here; but many facts and observations will be recorded, which may awaken some abler minds to the importance of codifying them and setting them forth for the benefit of mankind. Suffice it to say now, that out of 420 cases of congenital idiocy examined, some information was obtained respecting the condition of the progenitors of 359. Now, in all these 359 cases, *save only four*, it is found that one or the other, or both, of the immediate progenitors of the unfortunate sufferers had, in some way, widely departed from the normal condition of health, and violated the natural laws. That is to say, one or the other, or both of them, were very unhealthy or scrofulous; or they wre hereditarily predisposed to affections of the brain, causing occasional insanity; or they had intermarried with blood relatives; or they had been intemperate; or had been guilty of sensual excesses which impaired their constitutions.

Now, it is reasonable to suppose that, if more accurate information could have been obtained about the history of the other four cases, some adequate cause would have been found in them also, for the misfortune of the child, in the condition of the progenitors.

This subject of the hereditary transmission of diseased tendency is of vast importance; but it is a difficult one to treat, because a squeamish delicacy makes people avoid it; but if ever the race is to be relieved of a tithe of the bodily ills which flesh is now heir to, it must be by a clear understanding of, and a willing obedience to, the law which makes the parents the blessing or the curse of the children; the givers of strength, and vigour, and beauty, or the dispensers of debility, and disease, and deformity. It is by the lever of enlightened parental love, more than by any other power, that mankind is to be raised to the highest attainable point of bodily perfection.

Can there be so sad a sight on earth as that of a parent looking upon a son deformed, or halt, or blind, or deaf, with the consciousness that he himself is the author of the infirmity; or upon a sick and suffering daughter, fading and dying in early youth, from the gnawing of a worm which he himself placed within her breast; or a wayward and unmanageable child, urged and hurried on to lust, and licentiousness, and crime, by the irresistible force of passions which he himself bestowed upon it? If such parent erred in ignorance; if he had always obeyed the laws of life and morality, as far as he knew them, still must his suffering be grievous; but if he sinned against the clear light of God's law; if he secretly defiled the temple of his soul, ran riot in lust, fed the fire of passion until it burnt out the very core of his body, and then planted a spark from the smouldering ashes to shoot up into unhallowed flames in the bosom of his child—how horrible must be his sensations when he looks upon that child, consuming, morally, every day before his eyes! Talk about the dread of a material hell in the far-off future! The fear of that can be nothing to the fear of plunging one's own child in the hell of passion *here*. It is probable that there are thousands of such parents among us, who never dream that they are at all responsible for those bodily ailments of their offspring which sadden their own lives; or for the stupidity, the waywardness, or the vice which almost hardens their hearts against the children who manifest them, while, in reality, those ailments and vices are but the dregs of a poisoned chalice returned to their own lips.

It may be assumed as certain, that in all cases where children are born deformed, or blind, or deaf, or idiotic, or so imperfectly and feebly organized that they cannot come to maturity under ordinary circumstances, or have the seeds of early decay, or have original impetuosity of passions that amount to moral insanity—in all such cases the fault lies with the progenitors. Whether they sinned in ignorance or in wilfulness, matters not as to the effect of the sin upon the offspring. The laws of God are so clear that he who *will* read may do so. If a man violates them ignorantly, he suffers the simple penalty; if he violates them knowingly, he has remorse added to his suffering; but in no case can the penalty be remitted to him.

The conditions of the law of transmission of hereditary tendencies to disease of body and of mind are beginning to be known, but there are many circumstances which obstruct the spread of knowledge upon the subject. First and foremost among these is the mournful ignorance about Physiology. People are blind to principles which, if understood, would make the whole law clear and beautiful.

The transmission of any infirmity is not always direct. It is not always in the same form. It may be modified by the influence of one sound parent; it may skip a generation; it may affect one child more, and another less; it may affect one in one form, and another in another; and so, in a thousand ways, it may elude observation. But more especially does it escape observation, because it may affect a child merely by *diminishing*, not destroying, the vigour of his mind or body,—by almost paralysing one mental faculty, or giving fearful activity to one animal passion, and so reappearing in the child in a different dress from what it wore in the parent. Variety is the great law of nature, and it holds good in the transmission of diseased tendencies, as well as in everything else. But unerring certainty, too, is alike a characteristic of this law; and let no one flatter himself or herself that its penalties can be escaped.

The health and vigour of the body may be compared to a man's capital; it is a trust fund given to him by the Creator, of which he may expend the interest in the natural enjoyments of life, but he cannot encroach in the least on the principal without real loss. Every debauch, every excess, every undue indulgence, is at the expense of this capital. A rich man may throw away cents or dollars, and not feel it,—but he is really poorer for it; and a young man, with a large capital of health, may daily throw away part of it, and still feel strong; but every over-stimulant to the nerves, every overload to the stomach, is a cent or a dollar taken from his capital; feel it, or not feel it, he is poorer for it, and so will be the children afterwards born to him.

There is this difference, however, between the capital which God gives man, and that which he accumulates for himself,—that the one is never so great but its interest can be spent with enjoyment, while the other may be so enormous as to cumber and embarrass him like an overload of fat. He may grasp so much, that, like the boy with his fist full of olives in the narrow-mouthed jar, he cannot withdraw it, and will not let any drop.

Were it not for the action of certain principles which give to the race recuperative powers, there would be danger of its utter deterioration as a whole by the sins of so many of its individual members.

The conviction of the existence and the importance of the law of hereditary influences has been brought home so strongly by examining the condition of the unfortunate objects of this research, that this digression has been inevitable.

Before referring to the tabular views appended, we shall attempt to give an idea of the leading differences among the persons referred to, although it is no part of the object of this Report to establish a scientific classification of idiots. The best way, perhaps, to give an idea of the leading distinctive features of different classes of these unfortunate beings will be to describe several individual cases. For all humane and practical purposes, we may divide them into Pure Idiots, Fools, and Simpletons, —or Imbeciles, as they are sometimes called.

According to M. Séguin, the type of an idiot is an individual who "*knows nothing, can do nothing, cannot even desire to do anything.*" This is the maximum of idiocy; the minimum of intelligence; and but very few cases can be found (we were inclined to think none could) in which a being in human shape is so much below even insects, and so little above a sensitive plant. The vast European hospitals, in which the two ends of humanity seem to meet—where beneficence, guided by science, stoops to give attention to the most shocking and repulsive forms of human suffering and degradation;—those great lazar-houses of London and Paris do sometimes, as their records show, present such cases of idiocy as, one would fain hope, can be found nowhere else. But, alas! when, overcoming the repugnance to close contemplation of utter degradation, one looks carefully among the *sweepings* that are cast out by society for something that may be saved to humanity, he finds, even in our fair commonwealth, breathing masses of flesh, fashioned in the shape of men, but shorn of all other human attributes.

IDIOTS OF THE LOWEST CLASS ARE MERE ORGANISMS, MASSES OF FLESH AND BONE IN HUMAN SHAPE, IN WHICH THE BRAIN AND NERVOUS SYSTEM HAS NO COMMAND OVER THE SYSTEM OF VOLUNTARY MUSCLES; AND WHICH CONSEQUENTLY ARE WITHOUT POWER OF LOCOMOTION, WITHOUT SPEECH, WITHOUT ANY MANIFESTATION OF INTELLECTUAL OR AFFECTIVE FACULTIES.

FOOLS ARE A HIGHER CLASS OF IDIOTS, IN WHOM THE BRAIN AND NERVOUS SYSTEM ARE SO FAR DEVELOPED AS TO GIVE PARTIAL COMMAND OF THE VOLUNTARY MUSCLES; WHO HAVE CONSEQUENTLY CONSIDERABLE POWER OF LOCOMOTION AND ANIMAL ACTION; PARTIAL DEVELOPMENT OF THE AFFECTIVE AND INTELLECTUAL FACULTIES, BUT ONLY THE FAINTEST GLIMMER OF REASON, AND VERY IMPERFECT SPEECH.

SIMPLETONS ARE THE HIGHEST CLASS OF IDIOTS, IN WHOM THE HARMONY BETWEEN THE NERVOUS AND MUSCULAR SYSTEM IS NEARLY PERFECT; WHO CONSEQUENTLY HAVE NORMAL POWERS OF LOCOMOTION AND ANIMAL ACTION; CONSIDERABLE ACTIVITY OF THE PERCEPTIVE AND AFFECTIVE FACULTIES; AND REASON ENOUGH FOR THEIR SIMPLE INDIVIDUAL GUIDANCE, BUT NOT ENOUGH FOR THEIR SOCIAL RELATIONS.

Among idiots proper should be classed the following cases:—

E. G., aged eight years. This poor creature may be taken as a type of the lowest kind of idiocy. He has bones, flesh and muscles, body and limbs, skin, hair, &c. He is, in form and outline, like a human being, but in nothing else. Understanding he has none; and his only *sense* is that which leads him to contract the muscles of his throat, and swallow food when it is put into his mouth. He cannot chew his victuals; he cannot stand erect; he cannot even roll over when laid upon a rug; he cannot direct his hands enough to brush off the flies from his face; he has no language—none whatever; he cannot even make known his hunger, except by uneasy motions of his body. His habits of body are those of an infant just born. He makes a noise like that of a very sick and feeble baby,—not crying, however, in a natural way. His head is not flattened and deformed, as is usual with idiots, but is of good size and proportion.

It would seem as if the powers of *innervation* were totally wanting in him. There is no nervous energy; nothing to brace the muscles; no more power of *contractility* than in a person who is dead drunk. The involuntary muscular motions are properly performed; that is, the organic life goes on regularly; the heart contracts and dilates; the peristaltic motion of the bowels is regular.

The probable causes are hereditary ones. The grand-parents were very scrofulous and unhealthy. The parents were apparently healthy, but gave themselves up to excessive sensual indulgence. They lost their health in consequence of this, and were so well aware of it as to abstain and to recover again. In the mean time, five children were born to them, two of whom were like E. G., and died at five or six years of age: two others were very feeble and puny, and died young.

A male, aged nine years. This organism in the human form is hardly a grade higher than the preceding. He has no muscular contractility; he cannot stand, nor sit upright, nor even turn over; for, if laid upon his stomach, he paws and kicks until turned over upon his back, which position he likes best. He has not even power to masticate his food, though he swallows very well when it is thrust into his mouth. He has no language, but seems to understand some simple sentences. He has more intelligence than the boy above-named, and the principal trouble seems to be want of contractility. He can feel flies that alight upon his skin, and can brush them off. His habits are like those of an infant. His head is very small.

The causes are probably hereditary, and he seems to be the last and lowest of a constantly degenerating breed. The grand-parents were intemperate and depraved. The children born unto them were puny and weak-minded, and they sank still lower in the slough of vice and depravity. The mother of this boy was herself a simpleton; and this was her second illegitimate child. Though of feeble health, she gave herself up to excessive licentiousness, her passions becoming almost maniacal.

H. W., aged seventeen. This wretched being seems to be, like the preceding ones, so deficient in nervous energy that he lies almost as powerless as though he were a mass of jelly, without a bone or a muscle in his composition. If his legs are pinched or irritated, he seems to try to move them, but scarcely draws them up an inch. If flies alight upon his face, he can hardly reach them with his hand. He sometimes rolls his head from side to side with a languid motion, and this is the most he can do in that way, for he cannot raise it up even to take food. He is fed like a sick infant, with half-chewed victuals, from a spoon. He has no speech, and apparently no knowledge of persons. When food is brought near to him, something like a smile comes over his countenance; perhaps he is made aware of it by the smell.

His head is not very small, nor is it deformed. The family of which he comes is very scrofulous and degenerate physically.

His relatives (especially his mother) are, many of them, remarkable for erysipelatous humours, tumours, carbuncles, &c. One of his cousins is idiotic, though not of so low a degree as he is.

It is remarkable, that in this case, as well as the two preceding, there is not the peculiar *look* so common with idiots, and which may be better expressed by the word *monkeyish* than any other. When the animal nature is pretty active, and there is, at the same time, a governing intellect, the resulting expression is human. The higher the intellectual endowment, the more lofty and noble is the look; the lower the degree of endowment, the more nearly the look approaches that of animals, until we get down to the mere twinkle of cunning in the low rogue, or the monkeyish looks of the idiot.

Now, the three persons above mentioned do not seem to be idiotic from any deficiency in the size, or deformity in the shape or structure, of that part of the organization on which the manifestation of intelligence immediately depends. There is, at any rate, no appearance of anything of that kind; but there seems to be a want of *power* in that part of the organization by which the nervous fluid gives energetic action to the frame. The look is that of languor rather than that of idiocy.

Among idiots of the lowest class are found some who, unlike the preceding, seem to have a superabundance of *innervation*, who have great *muscular contractility*—that is, great command of all the muscles by the nervous system—and who are consequently very active. They appear like insane persons in a state of excitement, and yet they have no speech, and no reasoning faculties. The distinction made with so much ingenuity by a celebrated French writer holds true here—"*The insane man reasons falsely; the idiot reasons not at all.*"

Jonas ——, aged eight years. His body is well-proportioned and strong, but very small. His face has the deformed look of idiocy. The sides of his head seem to be at a fever heat. He is almost all the time in violent motion. His appetite is not only voracious, but evidently morbid and insatiable; for, after eating heartily at table, he swallows anything he can lay his hands upon—raw potatoes, the bark of trees, chips of wood, and even small stones. He has been known to swallow pebbles as large as chestnuts. He hears and seems to understand the meaning of some sounds, but has no speech. He has no sense of propriety, no affection, no attachment; his brothers and sisters are no more to him than the dog and cat.

His father was intemperate to the last degree. His mother was of a very scrofulous habit of body.

Cases of this kind are not very frequent, and they are often mistaken for cases of insanity. They are generally proper subjects for instruction, though the long continuance of their life is not probable, for there seems to be morbid action in the brain.

### FOOLS

MAKE THAT CLASS OF IDIOTS WHO HAVE THE MUSCULAR AND NERVOUS SYSTEM WELL DEVELOPED; POWERS OF LOCOMOTION AND ANIMAL ACTION; IMPERFECT SPEECH; PARTIAL DEVELOPMENT OF THE PERCEPTIVE AND AFFECTIVE FACULTIES, BUT VERY FEEBLE POWERS OF REASON.

This class is more numerous than the preceding. Cases are found in every town, in almost every almshouse. The type of this class would be a man who uses all his senses; who observes things about him; who can make simple sentences, and understand simple directions; but who obeys every animal impulse without any thought about responsibility to others, or consequences to himself.

The description of some of these cases will be put in such a form as to give an idea of the course that was followed in inquiring into the condition of these unfortunate persons.

It was obviously necessary to have some regular series of questions, or rather a series of subjects about which questions were framed upon the spot, and put in such form as the occasion and circumstances demanded.

Some of the terms used, as will be seen, are borrowed from a system of mental philosophy, which (however undeniable its claims are to have presented the clearest and best analysis of the human faculties ever yet known,) has not been relied upon by the Commissioners in their examination. In speaking of the instinct to oppose and destroy, of the sentiment of self-esteem and love of approbation, the faculty of number, &c., as manifested in the following cases, no reference is had to the question whether there is or is not a proportionate development of those parts of the brain which some able anatomists and keen observers of nature maintain to be the part of the organization which is most immediately instrumental in the manifestation of such instinct, sentiment, or faculty. Indeed, in most cases, the notes were taken before the actual measurements were made. It was thought, however, that the close personal examination of so many idiots presented too rare and important an opportunity for ascertaining their craniological as well as other bodily peculiarities to be lost; and accordingly it was improved, and the general results may be found in the Tables. It may be stated here, in general terms, that the result of this examination and measurement shows that no dimensions of the head, except extreme diminutiveness, and no shape whatever, can be relied upon as criteria of idiocy. A few of the worst cases of idiocy are those in which the head is normal as to size and shape. Nevertheless, the Tables show that, taking the aggregate of all the cases, an obvious relation is seen between the size and development of the cranium, and of its different parts, and the amount of intellectual power, and of the different kinds of mental manifestation.

The results of the observations and measurements are published without any inference being drawn, in order that those who choose to examine and study them may do so.

Some writers have hastily concluded, that because a few idiots, whose heads were smaller than the measure which had been laid down as the minimum of brain by which intelligence could be manifested, have nevertheless been partially educated; and because many others, with heads of normal size and shape, are hopelessly idiotic,—therefore the doctrine of the dependence of mental manifestation upon the structural condition of the brain is overthrown. They say, it has been asserted that persons with heads of a certain size must necessarily remain idiots, and they triumphantly point to certain idiots who have recently been trained to show a certain amount of intelligence, though their heads were smaller than this arbitrary standard.

This conclusion, however, does not seem justified by close and candid observation. *Size* is only *one* of the structural conditions of the brain upon which mental manifestations depend;—*quality* of fibre, health, exercise, &c., are others essentially modifying it. It may very well be that one anatomist and philosopher, who wrote fifty years ago, saying that a man with a head below a certain measurement must necessarily remain an idiot in spite of any means of education *then known*, would be still right in his general conclusions, notwithstanding means are *now* discovered to educe considerable intelligence out of such a supposed idiot. The result of close and extensive observations of idiots has been strongly to confirm, not only the doctrine of the *volume* of brain being one important element in the means of manifesting mental power, but all the main doctrines of that school of philosophy which teaches that God gives us the body not merely as the handmaid of the soul, but weds and welds the two together in bonds of dependence that death alone can sever.

That philosophy has been aptly illustrated by comparing the body to a musical instrument, the soul to an invisible player. It is indeed so; and if the harp have a thousand strings, and they all be kept in tune, then the soul discourses sweet and varied music. But the idiot's body is a wretched thing, and its few strings are so sadly awry, that even in a seraph's hand it could give nothing but jarring and discordant sounds.

The whole of the success which has recently been gained, in attempts to improve the condition of idiots, has arisen from the adoption in practice of the principles of that much-ridiculed doctrine which teaches that the first thing to be done is to put the *instrument* in tune. Surely, then, the attempt to show what are the material conditions of the bodily instrument in such a number of idiots as have been examined by the Commissioners will not be condemned by candid observers, as such attempts made upon other classes of men have too frequently been.

That the different degrees of keenness and vigour with which different *manifestations* of mind can be made by different individuals, and by the same individual at different times, do, in some way, depend upon the original nature and the actual condition of *some part* of the bodily organization, none are now found foolish enough to deny; that they do depend, moreover, most immediately upon the structure and condition of the brain and nervous system, few will doubt; that there must be some peculiar corresponding outward signs by which the internal structure and condition of the brain and nervous system *may* be known by examination of the outward man, will not be questioned by sagacious observers of nature; that such examination, made upon an extensive scale, can lead to any but good results, will not be asserted by any but the few who think that modern observations should only be made to confirm ancient theories. If it is found that a certain condition of brain is an invariable accompaniment of a certain passion; if the condition is more marked when the passion is strong, less marked when it is weak, and unobservable when the passion is wanting; if, moreover, the condition changes with age, waxing and waning as the passion grows or declines,—then the inference becomes almost inevitable, that there is relation of cause and effect; then the external sign by which such internal structure and condition can be known is as much the natural language of the passion as a smile is the natural language of gladness. Now, to say that, because such signs have not yet been satisfactorily ascertained, therefore they never can be ascertained, and that the attempt to ascertain is impious or foolish, is just what it would have been a few years ago to say that, because a nebula never had been resolved, therefore it never could be resolved; that infusoria never had been seen, and therefore never could be seen; and that to turn a telescope to the sky, or the microscope to the water, was impious and foolish.

But however certain it is, first, that the activity and strength of mental manifestations must depend upon the internal structure and condition of the bodily organization; and second, that this structure and condition, like everything material, must have signs and language,—no reference is had to such signs in the following cases.

When it is said that a certain idiot's instinct to fight and destroy is very active, no reference is had to the fulness of his head about the ears; it is meant simply that he strikes, bites, scratches, or smashes things, and thus proclaims, *in another kind of language*, the activity and strength of the propensity. In order to see how many cases there are of coincidence between the craniological development and the existence of the propensity, reference must be had to the Tables.

W. C., a lad aged thirteen years. BODILY AND MENTAL CONDITION OF PARENTS.—The father is a man of scrofulous temperament, and very puny and feeble both in body and mind. Has been insane at times, especially at religious revivals, at which he prays and exhorts.

The mother is of a similar habit of body and mind, and has always been considered a simpleton.

They have one other child, a girl aged twenty, who is a simpleton.

FUNCTIONS OF ASSIMILATION, DIGESTION, GROWTH, &c.—These seem to be pretty active and healthy. He is of the ordinary size, and, though subject to fits when enraged, he has tolerable health.

MUSCULAR VIGOUR, rather below the average.

APPETITE FOR FOOD is insatiable. Unless restrained, he will always so overload his stomach as to bring on fits. He is now limited to a certain ration, which is about double the quantity consumed by other boys of his age. His thirst is also insatiable. He has been known to drink six quarts of water in twenty-four hours.

INSTINCT OF REPRODUCTION does not manifest itself, for he has been carefully watched in this respect.

INSTINCT TO FIGHT AND DESTROY is pretty active. He not only defends himself by striking and scratching, but will rush at things and persons, and push them over. He pulls things to pieces, but does not seem to know how to use his fists to strike, or to handle a stick.

DISPOSITION TO HIDE AND CONCEAL is apparent in the manner in which he disposes of things.

DISPOSITION TO POSSESS AND HOARD is manifested by his claiming his own chair, and his own cup and plate at table; also by carrying apples and fruit to his room, to put them away.

SELF-ESTEEM is not apparent in any of his actions.

LOVE OF APPROBATION is feebly manifested.

GENERAL ACTIVITY OF SENSES.—The five senses are normal, though not acute, except smell.

PERCEPTION OF INDIVIDUAL OBJECTS is feeble. He knows those immediately about him, and the common household things, but he evidently does not know how to recognise persons and things as other children do.

PERCEPTION OF COLOUR unknown.

PERCEPTION OF NUMBER very imperfect; he could not tell the difference between two, three, four, and five.

PERCEPTION OF TIME feeble.

PERCEPTION OF MUSICAL SOUNDS null.

FACULTY OF LANGUAGE feebly developed. He knows a few words, but has no power to construct a sentence to express his wants. He hardly knows a hundred words.

CAUSATION he seems to have no sense of whatever. The nearest approach is his habit of stealing hot water and putting it away to cool, in order to gratify his thirst.

DISPOSITION TO IMITATE very feeble; he will pick up chips when he sees other persons doing so, but cannot understand a direction to do so.

BENEVOLENCE utterly wanting: the same with Veneration, Imagination, Conscience, Hope of the Future, &c.

Male, aged twenty-four. BODILY AND MENTAL CONDITION OF PROGENITORS.—The mother was a very intemperate prostitute, and not much else is known of her, except that she died of *delirium tremens*.

The father is rather apocryphal.

FUNCTIONS OF HIS GENERAL DEVELOPMENT AND CONDITION OF BODY, imperfect. Head is very small. The extremities are shortened at the end; that is, the bones of the hands, fingers, and feet are very short in proportion to the other bones, as if the central formative power had not been vigorous enough to push the growth to the circumference. He is scrofulous, and often covered with sores, scabs, &c.

FUNCTIONS OF ASSIMILATION, DIGESTION, GROWTH, &c., are pretty efficient.

MUSCULAR VIGOUR seems nearly equal to the average. When sufficient motive is held out, he can do hard work; but the will is wanting, because the nervous energy is wanting.

APPETITE FOR FOOD is healthy as to quality of what he eats, but ravenous as to quantity.

INSTINCT FOR REPRODUCTION is fiercely active and ungovernable, and leads him on blindly to excesses of various kinds. The instinct to fight and destroy seems manifested by his instantly resorting to force to destroy whatever opposes his will—to smash an inanimate object; to kill an animate one, whether it be a fly, a dog, or a child.

Aged twenty-two. The mother of this idiot was a very scrofulous and puny person; she was insane during her gestation with him, and died of consumption soon after his birth. She had three children. One was a simpleton, and died young. The other, a sister, is almost idiotic.

The father, a healthy man, married a healthy woman for his second wife, and has five healthy and intelligent children by her.

The head of this idiot is exceedingly small, measuring only 17·5 inches in its greatest circumference, 22 inches being the standard.

The other physical peculiarities need not be referred to here. His language is imperfect, like that of a little child. He understands all simple directions given in sentences short as his own.

There is a useful lesson to be learned from this poor youth's history and treatment. He was formerly very irritable and violent when enraged, breaking and destroying things. For this he was treated in the usual way: force was met by force. He was whipped and punished corporally in various ways, for every offence, by any one about him. As he grew older and stronger, the number of those who could whip him with impunity grew less, till at last the father was obliged to become executioner-general, and in the evening gave him a sound drubbing for the divers and sundry misdemeanours of the day. The father spared not the rod, but healed not the child, who, on the contrary, grew worse and worse. The lessons in punishment were not lost upon him. Whatever object offended him, he would beat and punish just as he had been punished. If it were a tool of any kind, he would smash and break it in pieces; if it were a dumb beast, he would beat and abuse it. He smashed rakes, hoes, &c., without number, and one day broke a cow's leg with an axe.

It happened one evening that a zealous member of the Peace Society was a visitor at the house, and witnessed a scene of contest in which the father barely came off victor. The visitor urged the father to follow a different course with his unfortunate son; to abandon all blows, all direct use of force, and try mild measures. By his advice, Johnny was made to understand that, if he should commit a certain offence, he would be mildly and kindly remonstrated with, have nothing but bread and water for supper, and be obliged to lie upon the floor, with only a little straw under him. Very soon he began himself to practise this mode of punishment upon the cattle. If the cow offended him, instead of flying into a passion and beating her, he addressed her gravely, telling her the nature of her offence, and assuring her of the consequences. He would then lead her out, lay some straw upon the ground, bring a little water and a crust of bread, and tell her that was all she could have for supper. One day, being in the field, he hurt his foot with the rake, and instead of getting angry as he was wont to do, and breaking the instrument to pieces, he took it up mildly but firmly, carried it home, got some straw, and laid the offending tool upon it; then he brought some bread and water, and demurely told the offender that it had been very naughty—that he did not want to hurt it—but it should have no other supper, and no bed to lie upon.

By such means he has been very much improved, not only in behaviour, but in temper. He is growing less violent and more manageable every day.

This is not at all strange; it is not even different from what happens every day with common children. The poor idiot could not understand much of the spoken words by which *reason* manifests itself, but he could understand the *natural language* of all the passions very well; the angry looks, the harsh voice, the threatening gesture, were felt in the full force of their meaning, and they roused in him the answering feelings of fear, rage, or revenge. These feelings, being called into frequent action, grew more prompt and more fierce by every day's exercise, and would at last have come to be spontaneously and habitually active. But, by withdrawing from before his eyes the natural language of those passions in others, his own were no longer awakened.

As a fierce dog sleeps quietly amid the din of other sounds, but rouses up with defiant growl at the angry bark of another dog, so anger sleeps quietly in our nature, unmoved by anything except *the language of its kind* in another person, which language it understands and answers in a moment. We may make this, and other like passions, sleep so long and so soundly, that they will grow feeble, and even die out; or we may rouse them up so often that they cannot sleep, even when we will them to do so. The moral of this idiot's history will not be lost upon those whose passions became so restive before they were aware of their nature as to be a source of perpetual trouble in after-life, when the moral sense had become awakened to the necessity and the difficulty of self-control.

SIMPLETONS ARE THE HIGHEST CLASS OF IDIOTS, IN WHOM THE HARMONY BETWEEN THE NERVOUS AND MUSCULAR SYSTEM IS NEARLY PERFECT; WHO CONSEQUENTLY HAVE NORMAL POWERS OF LOCOMOTION AND ANIMAL ACTION; CONSIDERABLE ACTIVITY OF THE PERCEPTIVE AND AFFECTIVE FACULTIES, AND REASON ENOUGH FOR THEIR SIMPLE INDIVIDUAL GUIDANCE, BUT NOT ENOUGH FOR THEIR SOCIAL RELATIONS.

As the class of fools is much larger than that of idiots, so that of simpletons is much larger than that of fools. Indeed, it is very difficult to estimate their number, or to say what persons shall be included in it, for they can only be measured by a sort of sliding scale, with a standard adapted to different localities and conditions of society. A Russian serf, a Bavarian boor, might enjoy his sinecure office of citizen, and fill his narrow social circle, with a paucity of intellect such as would incapacitate a man for political rights or social relations in Massachusetts. So, among the inhabitants of the *least* intelligent and active village population of Massachusetts, a youth might be thought to be of tolerable capacity, be permitted to go to the polls, and even into society, who would be rated as a simpleton, and treated as such, in the active and bustling crowd of one of our thriving marts, where the weak sink down and disappear, and the strong alone live and thrive. And so it may be with regard to time; a century hence, the standard of intellect and of knowledge may be raised so high as to exclude from the polls, as simpletons, men equal to some of our generation who consider themselves qualified not only to be citizens, but to hold offices. Who would arrest such progress, provided no qualification but that of knowledge and virtue could ever be required!

The persons put down in this Report as simpletons, are those about whom there could be no doubt, even in this day and generation. They are persons the highest of whom should be considered unable to take any responsibility, to contract matrimony, or to vote. The latter tests, however, should never be applied by interested parties. Some of the simpletons in the list have been wheedled into matrimony, and the bond afterwards cancelled by authority, though nobody can tell how many continue unchallenged. Politicians, too, are sometimes as blind as lovers to the demerits of a head which can command a hand. Several cases have occurred where the taxes were paid for *simpletons*, and they voted—until the opposite party showed that they had a greater number of *fools* whom they could qualify and bring to the polls; and then the poor creatures, who had been used to violate the purity of the ballot and to defraud an election, were thrown aside in contempt.

It has been the aim to include in this Report none who could be considered by impartial persons as *compos mentis*. They are susceptible of great improvement, and could be made useful and reputable men, but they cannot be taught in common schools, or trained in the common way.

The following cases will serve as specimens:—

H. C. F., aged thirty-three. PARENTAGE.—His mother was extremely intemperate for several years before his birth; she continued to be so for years afterwards, and died of *delirium tremens*. Condition of father not known.

FUNCTIONS OF DIGESTION, ASSIMILATION, GROWTH, &c., seem tolerably well performed. His body is pretty well developed, and his health generally good.

Muscular vigour is impaired by a singular affection of his nervous system, which gives to him the *air*, *gait*, and *appearance* of *a drunken man!* He seems to have inherited from his mother a strong resemblance to her acquired habit of body. He trips and staggers in his walk, and frequently falters in his other motions. The nervous fluid seems to flow unsteadily from the brain, or to be frequently wanted; hence the motions of his muscles are suddenly checked, his jaw is arrested in the act of chewing, his lips in the act of speaking; or, if walking, and the stoppage is considerable, he stumbles, perhaps falls down. Sometimes he remains insensible for a minute or two, and is afterwards utterly unconscious of what passed. More often the command of one muscle, or of one side, is lost for an instant, and he is obliged to hitch and wriggle along with the others. Thus the poor creature drags himself about, a living monument of his mother's shame.

Appetite for food is almost insatiable, and he is very gluttonous. It is said that his mother used to give him rum when he was an infant.

Instinct of reproduction does not manifest itself in an unnatural degree.

Instinct to fight and destroy is not over-active. He does not desire to break things, as some idiots do, but he is ready to fight in self-defence.

Instinct to possess and hoard displays itself in his readiness to store up food.

Disposition to hide and conceal shows itself in the cunning with which he compasses his purpose of obtaining things to eat, and of shirking work.

Self-esteem is manifested in various ways.

Love of approbation is the sentiment most acted upon by those who have the charge of him. To secure the praise and flattery of others, he will do anything in his power.

General activity of the five senses is normal.

Perception of colour is about as usual.

Perception of the relations of numbers is very imperfect. He can count off, by rote, even to a hundred, but can scarcely tell how much two added to three will make.

Perception of time is feeble. He keeps step pretty well in walking, but is perplexed in estimating the passage of time.

Sense of musical relations feeble; he never attempts to sing.

Faculty of language is imperfectly developed. He knows the names of individual objects and persons and can use common sentences, but does not use involutions and complicated expressions.

CAUSALITY seems active in proportion to his other faculties. He can build a fire, wash potatoes, and put them to boil for breakfast, and do similar simple household acts.

DISPOSITION TO IMITATION is not so active as in most persons of his class. Provided he attains an object or an end, he does not seem to care whether he proceeds in the same way that others do or not. In some idiots, this disposition is very strongly marked.

BENEVOLENCE (so little manifested by most idiots) seems active in this man. He is very tender-hearted. His pity is easily excited. He gives away readily of whatever he has.

VENERATION is but feebly manifested. He cares little for his parents, or his elders and superiors—of course, nothing for God.

CONSCIENCE is feebly developed, and he cannot be governed by appeals to it. *Hope* reaches not beyond the things of this life: scarcely beyond the things of to-day.

A. B., woman, aged fifty-five, not a pauper. PARENTAGE, &c. —Her grandmother was insane, and finally became idiotic. Her mother and all her brothers and sisters are puny and consumptive. Her youngest sister is stunted in growth, and scarcely *compos mentis*.

FUNCTIONS OF ASSIMILATION, GROWTH, &c., are imperfectly performed. She is humpbacked and nervous.

MUSCULAR VIGOUR, below average; she is incapable of bearing much fatigue.

APPETITE FOR FOOD is natural as to quantity, but her taste has become perverted by use of tea, coffee, spices, &c.

INSTINCT OF REPRODUCTION apparently active, though great pains have been taken to prevent its development. Character in this respect good.

INSTINCT TO FIGHT AND DESTROY is manifested in the degree usual with children. She shows passion sometimes, and if injured retorts, and immediately assails the offender.

DISPOSITION TO POSSESS AND HOARD is not shown in its usual activity; for, though she is desirous of possessing and owning things, she cares not to retain them long.

DISPOSITION TO HIDE AND CONCEAL shows itself not only in regard to material objects of possession, but sometimes in hypocritical conduct. She will put on certain airs in order to conceal some purpose which she may have.

SELF-ESTEEM is very strongly manifested by its usual natural language. If her simple understanding could be convinced twenty times in a day that she is sadly deficient in everything of which people are usually vain, it would make no difference; selfesteem springs up again as elastic as ever, and makes her regard herself with great complacency.

LOVE OF APPROBATION is one of the most prominent traits in her character. To gain the attention and praise of others, she will do things that would otherwise be very disagreeable to her.

TENDENCY TO IMITATION is very strong indeed. She does things as she has seen others do them; imitates their actions; and nothing but their example wins her from continual repetition of the same thing, in the same manner that she once learned to do it.

THE GENERAL ACTIVITY OF THE SENSES is normal.

PERCEPTION OF INDIVIDUAL OBJECTS, within a certain range, is good. She recognises most of the individuals of the village, and common things about her; but then her circle is narrow, and beyond it she takes no notice of differences between individual objects.

PERCEPTION OF COLOURS is not vivid, but no striking want of power noticed.

PERCEPTION OF NUMBERS limited. With the assistance of *objects*, she can count a score or two, as the number in a pile of plates, the stitches on a knitting needle; but she cannot count or reckon abstractedly without the aid of objects. She can count, for instance, a pile of ten or fifteen cents, but cannot tell how many cents are in two or three half-dimes. She cannot make change, therefore, or reckon higher than ten, even with the aid of her fingers.

PERCEPTION OF TIME feeble; she can tell the hour by the clock, but without idea of measuring the lapse of time by it.

PERCEPTION OF MUSICAL SOUNDS is apparent in her. She sometimes hums a tune; but no fondness for music has been engrafted upon this capacity, which might have been done.

FACULTY OF LANGUAGE is not well developed; and her range of words is limited, though she can make simple sentences very well.

PERCEPTION OF CAUSATION is very feeble.

BENEVOLENCE AND CONSCIENCE are feebly manifested.

HOPE is very feeble; the horizon of her future is bounded by to-morrow.

The cases thus very imperfectly sketched, will serve to give an idea of the different classes of idiotic persons, and of the mode in which the inquiry into their condition was pursued. But they are strongly marked cases each of its kind, and it must not be supposed that all idiotic persons can readily be ranged in one or other of these classes. The highest of the lower class of Idiots can hardly be distinguished from the Fool; the least stupid of Fools can hardly be distinguished from the Simpleton; and the highest among Simpletons stand very near the level of hundreds who pass in society for feeble-minded persons, but still for responsible free agents. These latter, indeed, are looked down upon by the crowd; but, then, the crowd is looked down upon by tall men; and these, in their turn, are looked down upon by the few intellectual giants of each generation, who stand higher by the whole head and shoulders than the rest.

This view of the gradation of intellect should teach us not only humility, but humanity; and increase our interest in those who are only more unfortunate than we are, in that their capacity for seeing and understanding the wisdom, power, and love of our common Father, is more limited than ours, in this stage of our being.

It is thought best not to close this Report without alluding to some

### CIRCUMSTANCES OR CAUSES WHICH PREDISPOSE PERSONS TO IDIOCY.

This is a difficult subject, requiring more scientific research and accuracy than this Report can pretend to. Some facts, however, which have been observed, and some thoughts which have suggested themselves, may possibly be of use to others who follow in this field. When certain circumstances are noted as *preceding* idiocy, it is not meant that they certainly caused it; indeed, it is hard to say that any one cause or condition in a parent will produce idiocy in the offspring; nevertheless, a number of causes united may do it. For instance, take the case—

Wm. B., aged thirteen, which is one of idiocy of the lowest kind. This boy cannot walk alone, and can hardly creep about. Has no speech, though some of his natural signs can be understood. He cannot feed himself with a spoon, but can cram food into his mouth with his fingers. His head is very small. His intellect is almost null, and of course the affective faculties are not manifested.

In searching for accompanying circumstances which may throw light upon the probable causes, it is found that the father was a very intemperate man. This is not enough, for all intemperate men do not have idiotic children. His wife was related to him by blood, though not within the degree of first cousin; and still less was this a sufficient cause for the idiocy of the son. The wife's family was tainted with idiocy, her aunt having an idiotic child. We find, therefore, both intermarriage and idiotism in the family; but still this was not cause sufficient, because the parents of this boy had seven other children, all of tolerably good parts.

Looking at the mother's condition during gestation, it is found that, at an early period of it, she was several times very much agitated by terror and mental distress; that at a later one, she became ill, and had great difficulty in carrying her child to its full period; and finally, that her confinement was very long, protracted, and painful.

May it not be that these circumstances caused idiocy in this case, though they might not do so in ordinary cases, where the intemperance, or the intermarriage, or the tainted blood, or all of them, were wanting? May it not be, likewise, that *any one* of these circumstances occurring alone,—the intemperance, the intermarriage, the family taint, the fright, the illness, or the long and difficult parturition,—though it would not cause idiocy, nor have any very manifest effect, might, nevertheless, materially *diminish* what would otherwise have been the bodily and mental vigour of the offspring?

With this explanation, and with the understanding that *probability*, and not *certainty*, is aimed at, mention will now be made of some of the *immediate causes* of idiocy; among which by far the most prolific one is

THE LOW CONDITION OF THE PHYSICAL ORGANIZATION OF ONE OR BOTH PARENTS.

It is said by physiologists, that among certain classes of miserably-paid and poorly-fed workmen, the physical system degenerates so rapidly, that the children are feeble and puny, and but few live to maturity; that the grandchildren are still more puny; until, in the third or fourth generation, the individuals are no longer able to perpetuate their species, and the ranks must be filled up by fresh subjects from other walks of life, to run the same round of deterioration.

It would seem that startled nature, having given warning, by the degenerated condition of three or four generations, at last refuses to continue a race so monstrous upon the earth.

We see here another of those checks and balances which the exhaustless wisdom of God pre-established in the very nature of man, to prevent his utter degeneration. As the comet, rushing headlong towards the sun, is, by the very velocity which it gains, and which seems hurling it into the burning mass, carried safely beyond,—so a race of men, abusing the power of procreation, may rush on in the path of deterioration until, arriving at a certain point, a new principle developes itself, the procreating power is exhausted, and that part of the human family must perish, or regain its power by admixture with a less degenerate race.

It will be seen by the Tables, that by far the greater part of the idiots are children of parents one or both of whom were of scrofulous temperament, and poor, flabby organization. It is difficult to describe exactly the marks which characterize this low organization, but the eye of a physiologist detects it at once. Regarding it as a matter relating to the mere animal man,—if a farmer had swine, cattle, or horses, as inferior to others of their kind as many of these people are inferior to other men and women, he would pronounce them unfit to breed from. Such persons are indeed unfit to continue the species, for while they multiply the number, they lessen the aggregate powers.

In saying that such persons are generally scrofulous, the word is used in its popular sense, without any pretension to pathological accuracy. Indeed, it is difficult to give a correct idea of scrofula, because its symptoms are so manifold and so various. The class of persons to whom reference is made may be known by several signs. They do not stand erect and firm; they seem rather to be trying to hold their head and shoulders up by their muscles, than to rest firmly and gracefully poised upon the spinal columns and lower extremities.

Red and sore eyelids, turgid lips, spongy gums, swellings in the glands, liability to eruptions and diseases of the skin, mark this class of persons. The skin is generally fair; the muscles flabby; the hair is light,—seldom hard, crispy, and strong. They are not liable to fevers and violent inflammations, as others are; but, when unwell, nature relieves herself by sores, ulcers, eruptions, &c.

The peccant humours show themselves upon the surface in various ways—swellings and ulcerations of the glands, blotches, tetters, ringworms, rash, salt rheum, &c.

But it is not the surface alone that is affected; the internal tissues are often vitiated, and show their morbid tendencies by various affections, of which cancer is the worst.

Great pains have been taken to ascertain the physical peculiarities of the blood relatives of most of the idiots whose names are upon the list. In reading over the description of more than four hundred families in which idiots are found, one is struck with the great number of cases in which the affections abovenamed are found. A few cases will give a better idea than any general description can do:—

F. D., aged four and a half years. This child is a poor, puny, and scrofulous creature. Her head is very small, being only sixteen inches in circumference. She is quite idiotic, as might be expected with a head of such dimensions, upon a frame so weak and low-toned. She is very feeble in the spine; her right side is torpid, and right arm seems paralysed. Her family is very thriftless and dirty, and presents the spectacle, so rare in this country, of sharing their room with the pigs and poultry.

The father is afflicted with salt rheum and other humours, which seem to affect his whole system, and make him dyspeptic and wretched.

The mother is a feeble creature, whose skin is covered with eruptions. One of her children, sister of F. D., lately died from a virulent cancerous affection.

Abner and Palmyra H., a brother and sister, aged thirty-three and forty-three, both idiotic. Heads small. Bodies of feeble and flabby fibre. The bones of the extremities seem shortened—that is, out of proportion as to length, compared to the body. They are both afflicted with scrofulous humours and sores.

The man shows some of those remarkable signs, often manifested by idiots, of the instincts which one can suppose men would have in the undeveloped animal stated. When a boy, he had a passion *for burrowing in the earth like a rabbit.* He still, at times, will wander off into the woods, dig a hole as for a cellar, collect wood, and go on for days with this occupation, until discovered and brought home.

The general appearance of these idiots is said to be remarkably *like that of their parents when they were in their long drunken debaucheries.*

Both the parents were of unhealthy habit of body, troubled with scrofulous humours, St. Anthony's fire, *rum-sores* (as they are called), and other eruptions. All these natural impurities were made worse by intemperance in drink and depravity of life. By temperance, cleanliness, and careful observance of all the natural laws, they might have corrected the vicious humours of their bodies, lived pleasant lives, and been blessed with children to comfort their old age; but they chose to outrage nature in every way, and she sent them their punishment in the shape of those idiotic children.

Cynthia T., a girl of eighteen years old, idiotic. She was deformed at birth about the eyes and nose. She still shows the marks of a very scrofulous temperament. The bones of the hands and feet are shortened, and the ends seem as if they had been *gnawed* off. The upper edges of the frontal and parietal bones seem shortened, thus reducing the size of the upper part of the brain, or rather, perhaps, being reduced by its non-development.

Her parents, uncles and aunts, cousins, &c., are afflicted more or less with St. Anthony's fire, salt rheum, cancerous sores, &c.

Her father, as if his constitution was not corrupt enough, poisoned it still more by liquid fire. He has an *idiotic cousin*, who resembles C. T. in many respects.

In seeking for the causes which lead to this sad deterioration of families, it will be found that the most prominent and prolific is

### INTEMPERANCE.

By inspection of the Tables, it will be seen that, out of 359 idiots, the condition of whose progenitors was ascertained, 99 were the children of drunkards. But this does not tell the whole story by any means. By drunkard is meant a person who is a notorious and habitual sot. Many persons who are habitually intemperate do not get this name even now; much less would they have done so twenty-five or thirty years ago; and many of the parents of the persons named in the Tables have been dead longer than that time. A quarter of a century ago a man might go to his bed every night muddled and sleepy with the effects of alcohol, and still not be called an intemperate man.

By pretty careful inquiry as to the number of idiots of the lowest class whose parents were known to be *temperate* persons, it is found that *not one quarter* can be so considered.

The effect of habitual use of alcohol seems to be to *lymphatize* the whole bodily organization; that is, to diminish the proportion of the *fibrous* part of the body—to make the *lymph* abound in all the tissues. The children of such persons are apt to be of the scrofulous character above described; and *their* children are very apt to be feeble in body and weak in mind. Idiots, fools, and simpletons are common among the progeny of such persons. Thus, directly and indirectly, alcohol is productive of a great proportion of the idiocy which now burdens the commonwealth. If, moreover, one considers how many children are born of intemperate parents, who, without being idiots, are deficient in bodily and mental energy, and are *predisposed by their very organization* to have cravings for alcoholic stimulants, it will be seen what an immense burden the drinkers of one generation throw upon the succeeding. Many a parent, by habitual stimulus applied to his own nervous system, forms and fashions his child in such wise that he is more certain to be made a drunkard by the ordinary temptations of life than the child of a temperate man would be, even if living from his youth upward within the temptations of a bar-room

Probably the habitual use of alcoholic drinks does a great deal to bring families into that low and feeble condition of body alluded to in the preceding section as a prolific cause of idiocy.

There is another vice,—a monster so hideous in mien, so disgusting in feature, altogether so beastly and loathsome, that, in very shame and cowardice, it hides its head by day, and, vampyre-like, sucks the very life-blood from its victims by night; and it may perhaps commit more direct ravages upon the strength and reason of those victims than even intemperance,—and that vice is

### SELF-ABUSE.

One would fain be spared the sickening task of dealing with this disgusting subject; but as he who would exterminate the wild beasts that ravage his fields must not fear to enter their dark and noisome dens, and drag them out of their lair,—so he who would rid humanity of a pest must not shrink from dragging it from its hiding-places, to perish in the light of day. If men deified him who delivered Lerna from its hydra, and canonized him who rid Ireland of its serpents, what should they do for one who could extirpate this monster vice? What is the ravage of fields, the slaughter of flocks, or even the poison of serpents, compared with that pollution of body and soul, that utter extinction of reason, and that degradation of beings made in God's image, to a condition which it would be an insult to the animals to call beastly, and which is so often the consequence of excessive indulgence in this vice?

It cannot be that such loathsome wrecks of humanity as men and women reduced to drivelling idiocy by this cause, should be permitted to float upon the tide of life, without some useful purpose; and the only one we can conceive is that of awful beacons to make others avoid,—as they would eschew moral pollution and death,—the course which leads to such ruin.

This may seem to be extravagant language; but there can be no exaggeration, for there can be no adequate description even, of the horrible condition to which men and women are reduced by this practice. There are among those enumerated in this Report some who not long ago were considered young gentlemen and ladies, but who are now moping idiots,—idiots of the lowest kind; lost to all reason, to all moral sense, to all shame,—idiots who have but one thought, one wish, one passion,—and that is, the further indulgence in the habit which has loosed the silver cord even in their early youth—which has already wasted, and, as it were, dissolved, the fibrous part of their bodies, and utterly extinguished their minds.

In such extreme cases there is nothing left to appeal to—absolutely less than there is in the dogs and horses, for they may be acted upon by fear of punishment; but these poor creatures are beyond all fear and hope, and they cumber the earth awhile, living masses of corruption.

If only such lost and helpless wretches existed, it would be a duty to cover them charitably with the veil of concealment, and hide them from the public eye, as things too hideous to be seen; but, alas! they are only the *most* unfortunate members of a large class. They have sunk down into the abyss towards which thousands are tending. The vice which has shorn these poor creatures of the fairest attributes of humanity is acting upon others, in a less degree, indeed, but still most injuriously; enervating the body, weakening the mind, and polluting the soul.

A knowledge of the extent to which this vice prevails would astonish and shock many. It is indeed a pestilence which walketh in darkness, because, while it saps and weakens all the higher qualities of the mind, it so strengthens low cunning and deceit, that the victim goes on in his habit unsuspected, until he is arrested by some one whose practised eye reads his sin in the very means which he takes to conceal it—or until all sense of shame is for ever lost in the night of idiocy, with which his day so early closes.

Many a child who confides everything else to a loving parent, conceals this practice in his innermost heart. The sons or daughters who dutifully, conscientiously, and religiously confess themselves to father, mother, or priest, on every other subject, never allude to this. Nay, they strive to cheat and deceive by false appearances; for, as against this darling sin,—duty, conscience, and religion, are all nothing. They even think to cheat God, or cheat themselves into the belief that He who is of purer eyes than to behold iniquity can still regard their sin with favour.

Many a fond parent looks with wondering anxiety upon the puny frame, the feeble purpose, the fitful humours of a dear child, and, after trying all other remedies to restore him to vigour of body and vigour of mind, goes journeying about from place to place, hoping to leave the offending cause behind, while the victim hugs the disgusting serpent closely to his bosom, and conceals it carefully in his vestment.

The evils which this sinful habit works in a direct and positive manner are not so appreciable, perhaps, as that which it effects in an indirect and negative way. For one victim which it leads down to the depths of idiocy, there are scores and hundreds whom it makes shamefaced, languid, irresolute, and inefficient for any high purpose of life. In this way the evil to individuals and to the community is very great.

It behoves every parent, especially those whose children (of either sex) are obliged to board and sleep with other children, whether in boarding-schools, boarding-houses, or elsewhere, to have a constant and watchful eye over them, with a view to this insidious and pernicious habit. The symptoms of it are easily learned, and, if once seen, should be immediately noticed.

Nothing is more false than the common doctrine of delicacy and reserve in the treatment of this habit. All hints, all indirect advice, all attempts to cure it by creating diversions, will generally do nothing but increase the cunning with which it is concealed. The way is, to throw aside all reserve; to charge the offence directly home; to show up its disgusting nature and hideous consequences in glowing colours; to apply the cautery seething hot, and press it on to the very quick, unsparingly and unceasingly.

Much good has been done of late years by the publication of cheap books upon this subject. They should be put into the hands of all youth suspected of the vice. They should be forced to attend to the subject. There should be no squeamishness about it.

There need be no fear of weakening virtue by letting it look upon such hideous deformity as this vice presents. Virtue is not salt or sugar, to be softened by such exposure, but the crystal or diamond that repels all foulness from its surface. Acquaintance with such a vice as this,—such acquaintance, that is, as is gained by having it held up before the eyes in all its ugliness,—can only serve to make it detested and avoided.

Were this the place to show the utter fallacy of the notion that harm is done by talking or writing to the young about this vice, it could probably be done by argument—certainly by the relation of pretty extensive experience. This experience has shown that, in ninety-nine cases in a hundred, the existence of the vice was known to the young, but not known in its true deformity; and that, in the hundredth, the repulsive character in which it was first presented made it certain that no further acquaintance with it would be sought.

There is one mode of treatment, however, often recommended by physicians, which in many cases deserves only denouncement as erroneous or sinful—that is, causing the victim to contract matrimony. The cure is generally effectual, and the mode in which it is accomplished may, in some cases, be justifiable; but certainly, in many others, the retribution of offended nature is awful, and seems like a whole volume of revelation of God's purpose. In no less than ten cases which are here recorded, the idiocy of the children was manifestly attributable to this sin of the parent. Now, if a cause which would be so carefully concealed, is brought out in these ten cases, in how many more must it have been at work unnoticed and unsuspected! And if these ten *extreme* cases of idiocy have been the visitations upon the children of the sins of the parents, how many times ten cases must there be where the visitation is less severe, but still awful! How much bodily disease and weakness; how much mental obliquity and imbecility; how much of ungovernable lust, are thrown upon the children of this generation by the vices of their fathers and mothers of the foregoing one!

There is one remarkable and valuable fact to be learned respecting this vice, from observation of idiots, and that is, that some of them, though they have no idea of right and wrong, no sense of shame, and no moral restraint, are nevertheless entirely free from it. They could never have been in the practice of it, else they would never have abandoned it.

From this may be inferred, that it is a pest generally engendered by too intimate association of persons of the same sex; that it is handed from one to another like contagion; and that those who are not exposed to the contagion are not likely to contract the dreadful habit of it. Hence we see that not only propriety and decency, but motives of prudence, require us to train up all children to habits of modesty and reserve. Children, as they approach adolescence, should never be permitted to sleep together. Indeed, the rule should be—not with a view only to preventing this vice, but in view of many other considerations—that after the infant has left its mother's arms, and become a child, it should ever after sleep in a bed by itself. The older children grow, and the nearer they approach to youth, the more important does this become. Boys even should be taught to shrink sensitively from any unnecessary exposure of person before each other; they should be trained to habits of delicacy and self-respect; and the capacity which nature has given to all for becoming truly modest and refined, should be cultivated to the utmost. Habits of self-respect, delicacy, and refinement with regard to the person, are powerful adjuncts to moral virtues; they need not be confined to the wealthy and favoured classes; they cost nothing; on the contrary, they are the seeds which may be had without price, but which ripen into fruits of enjoyment that no money can buy.

### INTERMARRIAGE OF RELATIVES.

In assigning this as one of the remote causes of idiocy, it is not meant that, even in a majority of cases, the offspring of marriage between cousins, or other near relations, will be idiotic. The cases are very numerous where nothing extraordinary is observable in the immediate offspring of such unions. On the other hand, there are so many cases where blindness, deafness, insanity, idiocy, or some peculiar bodily or mental deficiency, is seen in such offspring, of the first *or second* generation, that one is forced to believe they cannot be fortuitous. Indeed, the inference seems to be irresistible, that such intermarriages are violations of the natural law, though not such flagrant ones as always to be followed by *obvious* and severe punishment. If two full cousins, who are both in good health, and free from any marked predisposition to any disease or infirmity, should marry, the probability is, that their immediate offspring will have tolerably good constitutions—though no one can say how *much less* vigorous in body and mind they are than would have been offspring born to either parent from marriage with some one of another healthy family. On the other hand, if a man in whose constitution there lurks a predisposition to any particular disease of body or mind, inherited from his *father's* family, should marry a daughter of his *father's* brother or sister, there would be a strong probability that the disease or infirmity would appear in the offspring; while the probability of such reappearance would be less if he married a healthy cousin by his *mother's* side, and still less if he married a person free from all unhealthy predispositions, who was not related to him at all.

It is seen by the Tables that, out of 359 cases in which the parentage was ascertained, 17 were *known* to be the children of parents nearly related by blood; but as many of these cases were adults, it was sometimes impossible to ascertain whether their parents, who are dead, were related or not before marriage. From some collateral evidence, we conclude that at least 3 more cases should be added to the 17. This would show that more than one-twentieth of the idiots examined are offspring of the marriage of relations. Now, as marriages between near relations are by no means in the ratio of 1 to 20, nor are even, perhaps, as 1 to 1000 to the marriages between persons not related, it follows that the proportion of idiotic progeny is vastly greater in the former than in the latter case—that is, taking this limited number of 400 for what little it is worth as data for calculation. Then it should be considered that idiocy is only *one* form in which nature manifests that she has been offended by such intermarriages. It is believed by some, that blindness, deafness, imbecility, and other infirmities, are more likely to be the lot of the children of parents related by blood than of others. If so—and it seems likely that it is—then the probability of unhealthy or infirm issue from such marriages becomes fearfully great, and the existence of the law against them is made out as clearly as though it were written on tables of stone.

The statistics of the 17 families, the heads of which, being blood relatives, intermarried, tells a fearful tale.

Most of the parents were intemperate or scrofulous; some were both the one and the other; of course, there were other causes to increase chances of infirm offspring, besides that of the intermarriage. There were born unto them *ninety-five* children, of whom FORTY-FOUR were idiotic, twelve others were scrofulous and puny, one was deaf, and one was a dwarf! In some cases, all the children were either idiotic, or very scrofulous and puny. In one family of eight children, five were idiotic.

### ATTEMPTS TO PROCURE ABORTION.

It appears that out of the idiotic persons examined, at least *seven* were probably made so by attempts, on the part of their mothers, to procure abortion. We say *at least* seven, because it is natural to suppose that, in most cases, every effort would be made to conceal the crime; in many cases the circumstances, even if generally known at the time, would be forgotten in the course of a few years, so that those who had the charge of an idiot twenty or thirty years of age would hardly go back to causes preceding his birth in giving to a stranger an account of the case.

If, then, with all these inducements for secrecy, and all these liabilities to forgetfulness, we find that seven out of about four hundred idiots were made so by attempts at abortion, the probability is very strong that others, whose history we do not know, were made idiotic by the same dreadful crime. Attempts are sometimes made by young women to conceal their shame by getting rid of their unborn proof of it; but, failing in this, they get married, and the child is idiotic, though all children born afterwards of the same parents are sound and healthy. Several cases of this kind are among those above alluded to. One woman had *seven* sound children, and another had *six*, born in wedlock, though the oldest child of each of them, upon whom abortion was attempted, was idiotic.

This subject is indeed most painful. It is horrible to think that a mother should aim a blow at the life of her unborn babe, and, failing of murder, wound and maim his soul, and bring forth a drivelling idiot to be a life-long witness against her crime. But such is one of the forms in which the fruit of sin reappears to punish the sinner and forewarn all beholders.

There is nothing which nature so carefully guards as the *life* of her creatures. This must be secured, if necessary, at the expense of everything else. This care is manifested from the first moment of conception. The tender being, hidden in the innermost and vital centre of its mother, floating in an elastic fluid, and carefully enveloped, fold within fold, by curious membranes, is not only beyond her reach, but almost beyond the reach of accident. She may fall—her bones may be broken—she may be wounded even unto death—and her babe be still safe. She may, it is true, affect its health by her own intemperance in food or drink; she may affect its passions by indulging her own; but still it lives.

Now, the attempt to destroy what nature so carefully guards is a most dangerous one; and it can only succeed by using medicines or measures of such violence that the whole system is shaken to its centre, and the life of the mother put in peril in order to kill the babe. The attempts, however violent, may fail; they do fail, perhaps, oftener than they succeed; but, alas! the poor innocent who has escaped murder has not escaped injury. It cannot be doubted that many are made idiotic, and more have their faculties impaired and their bodies injured, by attempts at this unnatural crime.

Sceptical persons may naturally inquire how it is possible for the Commissioners to procure any reliable information concerning matters of this kind, since the parties would not be likely to criminate themselves. It is to be recollected, however, that most of such persons are very ignorant and indiscreet; that some of them do not perceive any guilt in an attempt to destroy evidence of shame; that women are very communicative; and that an inquisitive person, whose object was evidently only to learn all he could about the idiotic child, solely with a view to the good of that child, would obtain evidence not easily obtained from others.

Matters like these soon become known among the friends and neighbours of the parties, if they are of the ignorant class, and are spoken about without much reserve.

It may be said about this, as about supposed causes of idiocy referred to above, that great care has been taken to obtain evidence; that much has been suppressed which was deemed doubtful; and that the rest is given with such explanations of its source, that each one may place upon it as much reliance as he thinks it deserves.

We have thus alluded to some of the most obvious and fertile causes (either remote or proximate) of the existence of such a great number of idiots as are found in this, and all other countries called civilized. It would swell this Report to volumes, to examine these causes pathologically and minutely. Scientific research has not been our object; but we have sought diligently for every item and scrap of knowledge upon the subject of idiocy which could be of practical use to the Legislature. In so doing, we have been obliged, in some cases, to drag, as with a net, the lower depths of society, seeking for the pearls of truth. With those pearls there may be much worthless trash, but this will all perish, while the gems will remain indestructible; and if they are of value enough to redeem only one human being from the brutishness of idiocy, our labours will not be in vain.

